# Transport of the high-risk neonate

**DOI:** 10.1186/1824-7288-41-S1-A22

**Published:** 2015-09-24

**Authors:** Hubert Messner, Alex Staffler

**Affiliations:** 1 Department of Neonatology, Central Teaching Hospital of Bolzano, Italy

## 

Neonatal transport is continuously evolving and has developed to a cornerstone of modern perinatal medicine. Although the regionalization of perinatal care and delivery of high-risk neonates in appropriately designed centres improves neonatal outcome, neonatal transport represents an invaluable resource in order to guarantee tertiary level care throughout the region.

A highly trained, adequately skilled and well-equipped transport team is key in order to provide a good quality of care for a wide variety of clinical disorders and their potential complications. A safe and efficient neonatal transport begins in the referring hospital. An optimal communication between the referring team and the transport team is paramount. Besides collecting information the transport team may advise the referring team about specific steps to undertake until arrival [1].

Adequate resuscitation and effective stabilization improve survival and reduce the chances of deterioration during transport, especially in critically ill neonates with limited physiological reserves [2]. High risk conditions like pneumothorax, congenital diaphragmatic hernia, oesophageal atresia, cardiac malformations, abdominal wall and neural tube defects and hypoxic ischemic encephalopathy (HIE) are still life threatening and require highly specialized care and monitoring [3]. 

The equipment routinely used for monitoring in the neonatal intensive care unit (NICU) is not specifically designed for transport and may not function properly under transport conditions. However, standard monitoring including temperature, ECG, pulse oximetry and CO_2_-monitoring should be routinely provided [4]. 

Delivering adequate ventilatory support including the administration and measurement of iNO may be challenging. A ventilator with an integrated flow sensor is helpful to assess respiratory function. The recommended ventilatory strategies in the NICU like volume-targeted ventilation can minimize lung damage and maintain more stable paCO_2_ values. There is a strong physiological rationale supporting the use of pre-heated and humidified air in ventilated infants, since it reduces trauma to the respiratory epithelium and is helpful to stabilize the body temperature [5,6]. 

Pharmacologic management including catecholamines, prostaglandins and analgosedation via multiline infusions may be necessary, thus rendering difficult situations still more complex.

Active or passive body cooling in case of HIE is often demanding. Adequate protocols and an optimal temperature control to avoid accidental hyper- or hypothermia are essential [7]. 

In literature, data on high-risk transports are lacking, likely because of the difficulty to perform randomized studies in this field. Advanced training in resuscitation and stabilization of the neonate, as well as a specific transport and simulation based team training are mandatory for all personnel. 
